# Exposure to disinfectants (soap or hydrogen peroxide) increases tolerance to permethrin in *Anopheles gambiae* populations from the city of Yaoundé, Cameroon

**DOI:** 10.1186/1475-2875-13-296

**Published:** 2014-08-03

**Authors:** Christophe Antonio-Nkondjio, Marlene Youmsi-Goupeyou, Edmond Kopya, Billy Tene-Fossog, Flobert Njiokou, Carlo Costantini, Parfait Awono-Ambene

**Affiliations:** 1 Laboratoire de Recherche sur le Paludisme, Organisation de Coordination pour la lutte Contre les Endémies en Afrique Centrale (OCEAC), P.O. Box 288, Yaoundé, Cameroon; 2 Faculty of Sciences, University of Yaoundé I, P.O. Box 337, Yaoundé, Cameroon; 3 Institut de Recherche pour le Développement (IRD), UR 016, 911, avenue Agropolis, P.O. Box 64501, 34394 Montpellier cedex 5, France; 4 Vector group, Liverpool School of Tropical Medicine, Pembroke Place, Liverpool L3 5QA, UK

**Keywords:** *Anopheles gambiae*, Soap, Hydrogen peroxide, Permethrin tolerance, Selection

## Abstract

**Background:**

The rapid expansion of insecticide resistance is limiting the efficiency of malaria vector control interventions. However, current knowledge of factors inducing pyrethroid resistance remains incomplete. In the present study, the role of selection at the larval stage by disinfectants, such as soap and hydrogen peroxide (H_2_O_2_), on adult mosquito resistance to permethrin was investigated.

**Methods:**

Field *Anopheles gambiae sensu lato* larvae, were exposed to variable concentrations of soap and H_2_O_2_. Larvae surviving to acute toxicity assays after 24 hours were reared to the adult stage and exposed to permethrin. The susceptibility level of adults was compared to the untreated control group. The effect of soap or hydrogen peroxide selection on the length of larval development and emergence rate was assessed.

**Result:**

Larval bioassays analysis showed a more acute effect of hydrogen peroxide on mosquito larvae compared to soap. The regression lines describing the dose mortality profile showed higher mean and variance to hydrogen peroxide than to soap. The duration of larval development (<5 days) and adults emergence rates (1 to 77%) were shorter and lower compare to control. *Anopheles gambiae s.l.* larvae surviving to selection with either soap or hydrogen peroxide or both, produced adults who were up to eight-times more resistant to permethrin than mosquitoes from the untreated control group.

**Conclusion:**

The present study shows that selective pressure exerted by non-insecticidal compounds such as soap and hydrogen peroxide affect *An. gambiae s.l.* tolerance to pyrethroids. This requires further studies with regard to the adaptation of *An. gambiae s.l.* to polluted habitats across sub-Saharan Africa cities.

## Background

Insecticide-based interventions (IRS and LLINs) represent the main strategy for malaria prevention across sub-Saharan Africa [1]. However, the effectiveness of these control measures is threatened by the rapid expansion of insecticide resistance [2]. Identifying the sources of pyrethroid resistance is becoming crucial and adheres to WHO Global Plan for Insecticide Resistance Management [3]. The majority of insecticides currently in used in public health are also used as pesticides in agriculture. The fact that large quantities of these compounds are used as agrochemicals is considered to have favoured the emergence and spread of pyrethroid resistance [4-6]. In Cameroon, consistent with a strong selective pressure exerted by agricultural use of insecticides, high prevalence of insecticide resistance was reported in areas with large agro-industrial estates, extensive cotton cultivation or market gardening [4,5,7-9]. With the rapid expansion of unplanned urbanization in the cities of Douala and Yaoundé, DDT and pyrethroid resistance was also reported to be highly prevalent in *Anopheles gambiae* populations emerging from both agricultural cultivated sites and polluted sites [10,11]. Bionomic studies assessing the distribution of *Anopheles coluzzii* and *An. gambiae* formally known as molecular forms M and S respectively in the city of Yaoundé, revealed high tolerance of *An. coluzzii* present in the city centre to ammonia compared to *An. gambiae* highly prevalent in the city suburbs [12,13]. Whether physiological changes, which have contributed to shape the distribution of *An. gambiae* and *An. coluzzii* in the city of Yaoundé, is affecting mosquito tolerance to insecticides is still to be ascertained. However, the fast evolution of insecticide resistance in the city of Yaoundé suggests the possible implication of several factors responsible for generating selective pressure and requires further attention. In their natural habitats, mosquitoes are usually exposed to a large set of conditions which affect their fitness, development, distribution and level of susceptibility to insecticides [14]. Thermal adaptation was reported to increase *Anopheles stephensi* susceptibility to Plasmodium infection and tolerance to insecticides, such as carbamates and malathion [15,16]. Pre-exposure to several compounds including heavy metals, plant allelochemicals or petroleum products are also considered to induce in insects cross-resistance to insecticides [17-21]. Meanwhile despite the huge amount of disinfectants such as soap or hydrogen peroxide (H_2_O_2_) regularly eliminated in the environment their impact on mosquito populations bionomic has so far been poorly explored across sub-Saharan Africa. Hydrogen peroxide and different formulations of soap including commercial or antibacterial soaps were shown to have a larvicide effect against several pest insects or to be lethal to several organisms [22-24]. Soap is manufacture by mixing fatty acids to a salt (NaOH or KOH) but more formulations include active ingredients (antibacterial), which most of the time increase the larvicide effect [23]. This making that, new formulations of soap might even be more toxic against arthropods than previous [23,25]. Because of lack of appropriate drainage systems in sub-Saharan cities, the large majority of pollutants released by anthropogenic activities are eliminated in the environment and these compounds accumulate in rivers or stagnant water bodies. Despite their toxicity, their impact on the dynamic of the aquatic fauna and the natural ecosystem is poorly understood. In the current study, the role of selection at the larval stage by soap and hydrogen peroxide (H_2_O_2_) on adult mosquito resistance to permethrin was assessed.

## Methods

### Study sites

The study took place in Yaoundé (3° 51’N 11° 30’E), the capital city of Cameroon. The city is situated within the Congo-Guinean phytogeographic zone characterized by a typical equatorial climate with two rainy seasons extending from March to June and from September to November. The annual average rainfall in Yaoundé is 1,700 mm. The city is situated 800 m above sea level and is surrounded by many hills. Larval collections in Yaoundé were carried out in five districts situated in the city centre: Mokolo, Messa, Olezoa, Ahala and Combattant. The study was conducted under the ethical clearance N° 216/CNE/SE/09 delivered by the Cameroon National Ethics Committee Ref N° IORG0006538-IRB00007847-FWA00016054.

### Mosquito identification

Anopheline larvae were identified morphologically using the Gillies and Coetzee keys [26]. Mosquitoes belonging to the *An. gambiae* complex were subjected to PCR assays designed for species and molecular forms identifications [27]. Genomic DNA used for molecular analysis was extracted from larvae according to Cornel [28] protocols.

### Bioassay experimentations

#### Preparation of test solutions for larvae bioassays

Stock solutions and serial dilutions were prepared starting from commercial solutions of soap (lemon dish liquid, Colgate Palmolive Cameroon) and hydrogen peroxide (H_2_O_2_) (Laboratoires Gilbert, France) following the protocol described in WHO guidelines [29]. A stock solution at 5% was prepared by mixing the corresponding volume of soap or H_2_O_2_ to distilled water. Test concentrations were prepared by serial dilutions in distilled water to obtain a final test volume of a 100 ml.

### Experimental procedure of bioassays with larvae

Preliminary bioassays were conducted to assess the toxicity of soap or H_2_O_2_ on a susceptible strain (The Yaoundé laboratory colony consisting exclusively of *An. coluzzii* and known to be susceptible to pyrethroids). Following these bioassays a range of concentrations was then determined for assessing field larvae susceptibility.

**Figure 1:**
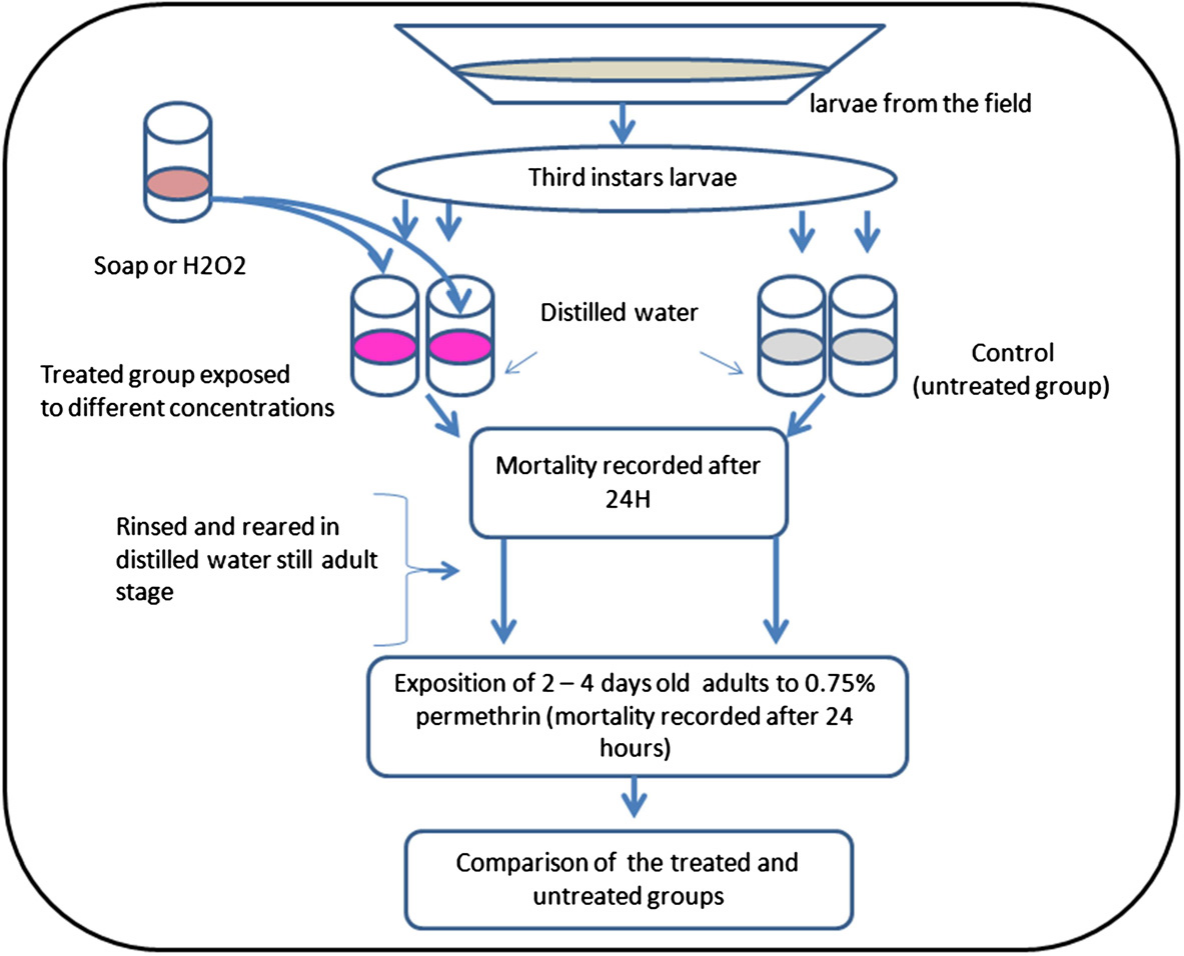
A graph describing the experimental design of the study.

Field collected *An. gambiae sensu lato (s.l.)* larvae, were divided in two separate groups the treated and untreated groups. The treated group consisting of third instars were exposed in 100 ml freshly prepared soap or H_2_O_2_ solution at the required concentration. At least seven different concentrations were tested and 10 to 20 replicates conducted. Control cups had 100 ml of distilled water. Batches of 25 to 30 larvae were distributed per cup. Larvae of each breeding site were exposed to all test concentrations. After an initial observation period of 2 hours in distilled water, larvae were transferred into test cups with the required xenobiotic concentration. Larval mortality was recorded after 24 hours exposure (Figure 1). Larvae were considered dead when they were incapable of any active movement when touched. The mortality rate was corrected by the formula of Abbott [30] if it was between 5% and 20%. The lethal concentration killing 50% and 95% of larvae (LC50 and LC90) was calculated. Mosquito of the untreated control group were placed in distilled water during the experiment.

### Adults susceptibility tests

Larvae surviving exposure to soap or H_2_O_2_ and larvae from the untreated control group, were reared in distilled water until the adult stage before being exposed to 0.75% permethrin. Insecticide susceptibility tests were performed with two- to four-day-old unfed *An. gambiae s.l.* females. Batches of 20 to 25 mosquitoes per tube were exposed to permethrin (0.75%) impregnated papers (supplied by Dr Wondji of, Liverpool School of Tropical Medicine, UK) for one hour. The number of mosquitoes knocked down by the insecticide was recorded every 5 minutes during exposure. After exposure, mosquitoes were fed with a 10% glucose solution and the number of dead mosquitoes was recorded 24 hours post-exposure. Tests using untreated papers were systematically run as controls. The mortality rates were corrected using the Abbot formula [30] whenever the mortality rate of the controls was between 5 and 20%. World Health Organization criteria [31] were used to evaluate the resistance and susceptibility status of the mosquito population tested. Three classes of insecticide susceptibility were defined: insecticide resistant (<80%), insecticide tolerant (80 to 97%), and insecticide susceptible (>97%).

### Statistical analysis

Using a log-probit regression analysis, the toxicity effect of H_2_O_2_ or soap on mosquito larvae mortality was computed and lethal concentrations LC50 and LC95 were determined using the software XLSTAT. The estimation uses the iterative method of maximum likelihood to fit a linear regression between the log of insecticide concentration and the probit of mortality. Comparison of the prevalence between groups was performed using chi square test. Estimates of odd ratio values between adult mortality rates and lethal concentrations used for larval selection were computed using the software MedCalc V11.5.0.0.

## Results

### Assessment of lethal concentrations of Soap and H_2_O_2_ on *Anopheles gambiae s.l.* larvae

**Figure 2:**
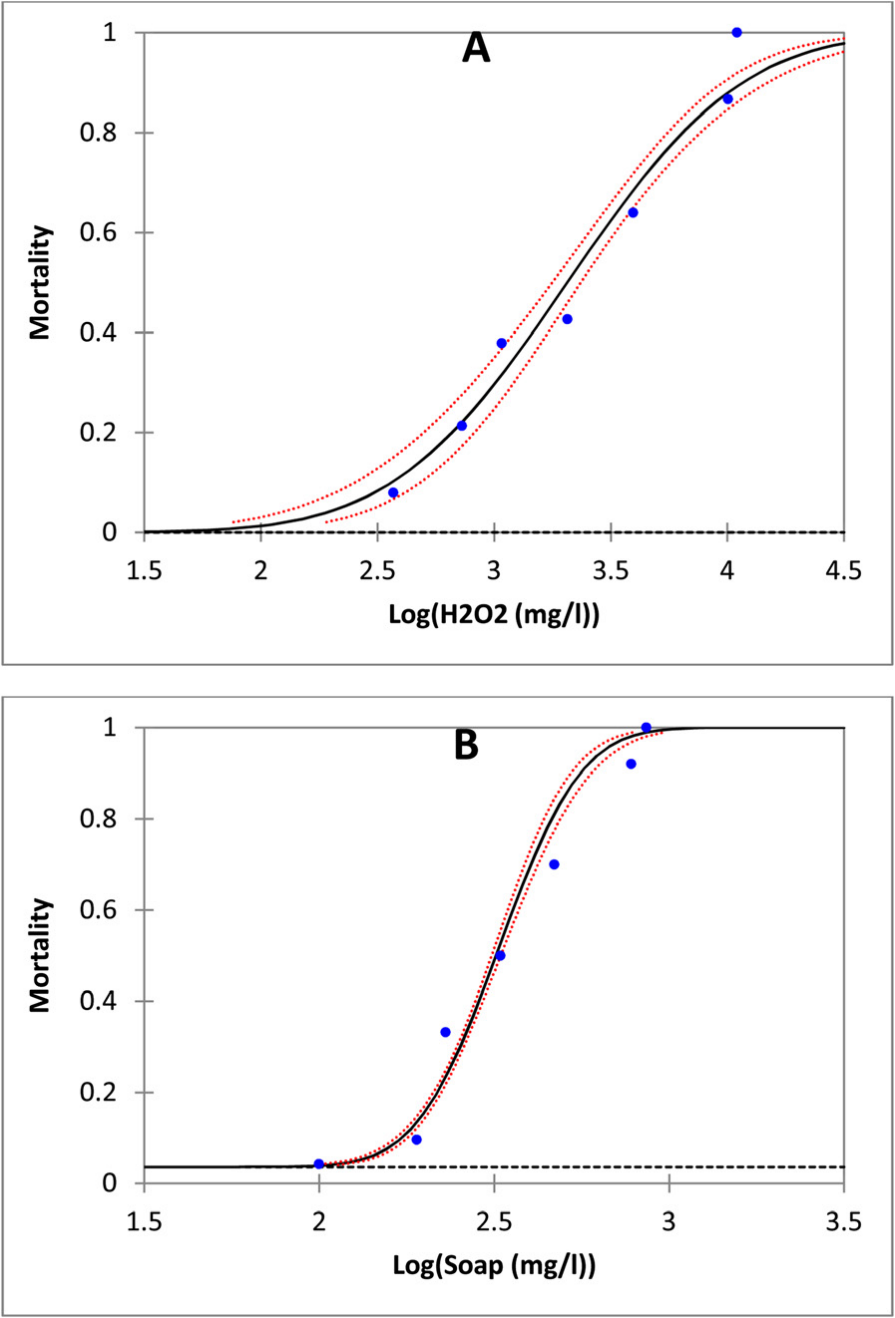
**Dose mortality curve for *Anopheles gambiae s.l. *larvae form Yaoundé exposed to hydrogen peroxide (H**_**2**_**O**_**2**_**) (A) and soap (B) for 24 hours.** Logistic regression line was fitted to dose response data using a XLSTAT 2013. In red dots the ±95% confidence limits.

A total of 7,204 field *An. gambiae* larvae were exposed to various concentrations of soap and 4962 to various concentrations of H_2_O_2_. A probit regression model was used to assess the effect of soap and hydrogen peroxide (H_2_O_2_) on third instars larvae mortality. A positive and significant correlation between larval mortality and log (dose) of soap or H_2_O_2_ was recorded (p < 0.001). The regression lines describing the dose mortality response of mosquitoes to soap or peroxide hydrogen, showed some differences in the distribution of individual tolerance thresholds to these two pollutants. Higher mean and variance was recorded with H_2_O_2_ than with soap (Figure 2). The natural mortality calculated by the model was respectively 3.6% for soap and 0% for H_2_O_2_ and was not significantly different from observed mortality in the untreated control groups (3.1% for soap and 1.8% for H_2_O_2_) (P > 0.4) and confirm that, no other factor was inducing larval mortality. The LC50 and LC95 lethal concentrations estimated are presented in Table 1.

**Table 1:** **Estimates of the lethal concentration doses (LC50 and LC95) of hydrogen peroxide (H**_**2**_**O**_**2**_**) and Soap calculated from generalized linear models assessing the effect of H**_**2**_**O**_**2 **_**and soap concentration (expressed in mg/l) on *Anopheles gambiae s.l. *** **larval mortality**

**Lethal Concentration (LC)**						
		**Soap**			**H**_**2**_**O**_**2**_	
Species	N	LC50 (95% CI)	LC95 (95% CI)	N	LC50 (95% CI)	LC95 (95% CI)
*An. gambiae s.l.*	7,204	320.1 (311.2 – 330.2)	651.3 (605 – 709.9)	4,962	2060.8 (1776 – 2353.9)	19059.9 (14903.7 – 26069)

N, Number of larvae tested; 95% CI, 95% confidence limits.

### Molecular identification of mosquito processed

In total, 197 specimens were analysed to determine species and molecular forms of the *An. gambiae* complex, three specimens were *An. gambiae* (S molecular form), the remaining 194 (98%) specimens were *An. coluzzii* (M molecular form).

### Chronic sub-lethal effect of selection by soap and H_2_O_2_ on *An. gambiae* development at the larval stage

Third instar *An. gambiae* larvae recorded as survivors after 24 hours exposure were rinsed and reared in distilled water until adult stage to assess the effect of selection by soap and H_2_O_2_ on larval development in laboratory conditions. Larvae pre-exposed to soap or H_2_O_2_, showed high emergence rate (41%, 77%) when exposed to lower concentrations while higher mortality was recorded with higher concentrations (Table 2). The average duration of larval development was reduced compare to control and varied from 4.43 to 4.96 days for larvae pre-exposed to soap and 4.33 to 4.89 for larvae pre-exposed to H_2_O_2_ (Table 2). A significant evolution of daily mortality compare to control (P < 0.001) was recorded after mosquito pre-exposure to H_2_O_2_ or soap.

**Table 2:** **Effect of pre-exposure to soap or hydrogen peroxide (H**_**2**_**O**_**2**_**) on *An. gambiae *** **larvae emergence rate and length of larval development**

		**Larvae exposed to soap**			**Larvae exposed to H**_**2**_**O**_**2**_	
	**N**	**Proportion of larvae emerging as adults**	**Average length in days of larval development (±95% CI)**	**N**	**Proportion of larvae emerging as adults**	**Average length in days of larval development (±95% CI)**
Control	424	82 ± 1%	6.24 ± 2.04	424	82 ± 1%	6.24 ± 2.04
LC10	562	77 ± 1.5%	4.96 ± 1.73	375	41 ± 2.4%	4.72 ± 1.7
LC20	470	38 ± 2%	4.43 ± 1.7	225	39 ± 3.1%	4.89 ± 1.72
LC30	548	12 ± 0.9%	4.78 ± 1.71	513	12 ± 0.9%	4.42 ± 2.19
LC50	761	3 ± 0.2%	4.44 ± 1.83	382	1 ± 0.1%	4.33 ± 2.1

N, Number of larvae; 95% CI, 95% confidence limits.

### Effect of larval tolerance to soap or H_2_O_2_ on adult *Anopheles gambiae* resistance to permethrin

**Figure 3:**
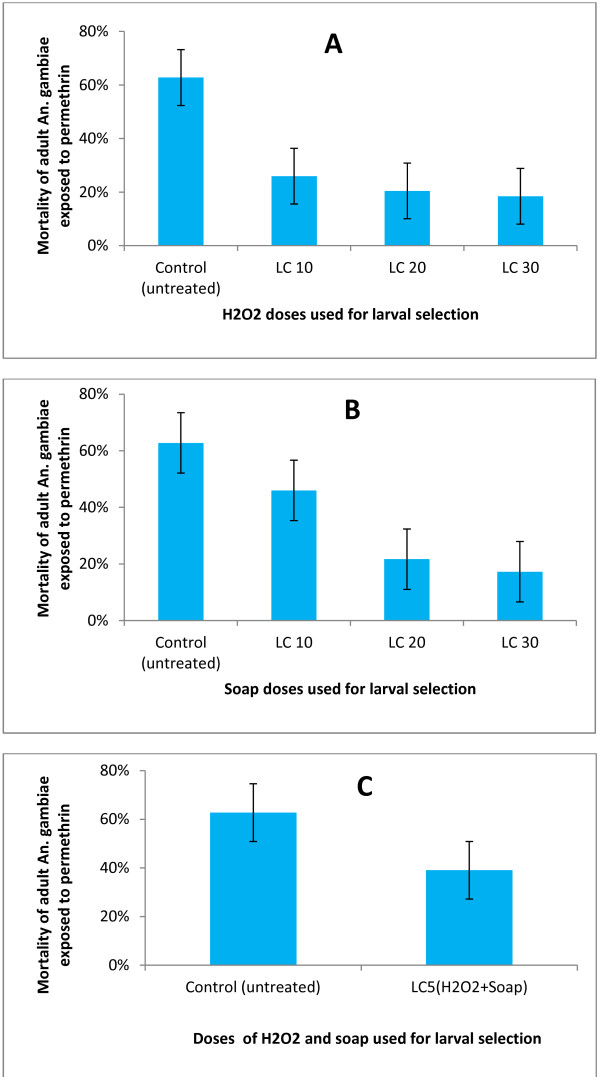
**Diagrams presenting adult *An. gambiae s.l. *** **mortality rate after one hour exposure to permethrin (0.75%) of mosquitoes pre-exposed during the larval stage to hydrogen peroxide (H**_**2**_**O**_**2**_**) (A) soap (B), or a mixture of soap and H**_**2**_**O**_**2 **_**(C) and control with ±95% confidence limits; LC represents lethal concentration.**

Larvae surviving at different concentrations of soap or H_2_O_2_ were reared to the adult stage and susceptibility to permethrin (0.75%) after one hour exposure using WHO standard bioassays was conducted [31]. Increased tolerance of mosquitoes pre-exposed to soap or H_2_O_2_ was recorded compare to the untreated control. A similar trend was recorded when mosquitoes were exposed to a mixture of soap and H_2_O_2_ at low concentrations (Figure 3). The level of tolerance at the adult stage appeared significantly dependant of the concentration of soap or H_2_O_2_ used for larval selection. Further analysis assessing the strength of cross resistance to either soap or H_2_O_2_ and permethrin showed that larvae expressing tolerance to higher concentration of soap or H_2_O_2_ produced adults who were seven to eight times more resistant to permethrin than the untreated control (Table 3).

**Table 3:** **Strength of association between selection of mosquitoes at the larval stage with different sublethal concentrations of soap or hydrogen peroxide (H**_**2**_**O**_**2**_**) and mosquito tolerance to permethrin 0.75% at the adult stage**

	**Comparison of mortality rates between groups**					
	**LC10 vs control**	**LC20 vs control**	**LC30 vs control**	**LC20 vs LC10**	**LC30 vs LC10**	**LC30 vs LC20**
Soap	1.98**	6.09**	8.09**	3.07**	4.08**	1.33 (NS)
H_2_O_2_	4.81**	6.58**	7.45**	1.37 (NS)	1.54 (NS)	1.13 (NS)

LC10, LC20, LC30 refer to sublethal concentrations of either soap or H2O2 used to select mosquitoes at the larval stage (LC10, LC20, LC30 concentrations leading to 10, 20 or 30% mortality rate); control , untreated group; NS, Non significant *, P < 0.05; **, P < 0.001.

## Discussion

Despite the rapid expansion of insecticide resistance in Cameroon cities [4,5,32], cross-resistance between non-insecticide chemicals and pyrethroids in mosquito populations has received until date little attention. In the present study, the relationship between *An. gambiae s.l.* larval survival to disinfectants respectively soap and hydrogen peroxide (H_2_O_2_) and resistance of adult mosquitoes to permethrin was investigated. The study reveals that mosquitoes exposed during the larval stage to soap or hydrogen peroxide were significantly more tolerant at the adult stage to permethrin than control (untreated group). The findings are consistent with previous studies indicating that thermotolerance or exposure to heavy metals or natural xenobiotics, is capable of increasing mosquito resistance to insecticides and/or detoxification genes expression [16,19,20,33]. Large variance in the tolerance level of field larvae to soap and H_2_O_2_ was recorded and could likely be associated to prior selection by pollutants present in natural breeding sites. Hydrogen peroxide and soap, are two compounds which presence in the nature is largely associated to anthropogenic activities. Hydrogen peroxide is known for its toxic effect for different living being [24]. Soap on the other hand is a detergent largely used in domestic activities. Commercial soap was demonstrated to be toxic to crickets, American cockroaches and mosquito larvae [34-36]. Soap has also been used as insecticide against different pest insects [25]. Because soap and hydrogen peroxide are oxidants or because they are capable of releasing toxic by-products, such as free radicals (hydroxyl, superoxide, or lipid peroxyl radicals), they could act by inducing oxidative stress which effect could be dramatic for mosquito larvae. It is likely that the low susceptibility detected in field larvae could be associated to increase tolerance to oxidative stress. It was demonstrated for *An. gambiae* that, oxidative stress genes such as oxidase resistance (OXR1) apart from protecting against oxidative stress, also regulates the basal level of catalase and glutathione peroxidase expression, two enzymes involved in the detoxification of hydrogen peroxide and several other xenobiotics [37]. Although neither *kdr* nor metabolic resistance assays were conducted, it is likely that these two mechanisms are responsible for the limited susceptibility of *An. gambiae* to permethrin. Assessments conducted between 2009 and 2012 in both Douala and Yaoundé, identified *kdr* as the main resistance mechanism in both cities (frequency > 60%) and metabolic resistance also implicated in Yaoundé [9,10,38]. It is probable that detoxification genes, such as CYP6P3 and CYP6M2 highly prevalent in DDT and pyrethroids resistant mosquito specimens emerging from polluted sites [9], could be involved in resistance to these xenobiotics. In the light of previous studies cuticular mechanisms (bioaccumulation of pollutants or reduced cuticular penetration) might have a limited role in xenobiotic or pyrethroid detoxification in mosquitoes originating from polluted or cultivated sites in Yaoundé [9].

Studies conducted so far in the city of Yaoundé, suggested different tolerance levels driving niche partitioning between populations of *An. coluzzii* and *An. gambiae*[13]. Higher ammonia tolerance display by *An. coluzzii* predominant in urban centres was considered as an adaptive trait to urban areas given that this toxicant occurred on average at higher concentration in urban larval habitats compared to rural ones, where *An. gambiae* predominates [13]. Yet no implication of this distribution on mosquito tolerance to insecticide has so far been established and both *An. coluzzii* and *An. gambiae* have been recorded resistant to pyrethroid [9,10]. Despite the fact that the sample used for the study consisted almost exclusively of *An. coluzzii* specimens, phenotypes with different levels of tolerance to soap and H_2_O_2_ were recorded and this probably highlights a process of microevolution leading at improving this species capacity of adaptation to new environmental conditions. Consistent with these observations, cytological analysis conducted on *An. coluzzii* and *An. gambiae* across Cameroon recorded different chromosomal arrangements induced by directional or balancing selection strongly correlated to prevailing ecogeographical conditions [39].

It also appeared from the study that selection with soap or H_2_O_2_ reduced significantly the proportion of larvae emerging as adults. The following suggest that tolerance to pollutants occurs at significant fitness cost as reported elsewhere [40]. It is possible that in the nature, because xenobiotics could be sequestrated or diluted in large water bodies, their impact on mosquito fitness might be attenuated. Further investigations might be necessary to capture the influence of the current adaptation of mosquito populations to polluted habitats on subsequent generation’s fitness.

## Conclusion

Because pyrethroid resistance limit the efficiency of control interventions, identifying compounds responsible for pyrethroid resistance emergence is becoming critical for improving control strategies. The present study, showed that alongside insecticide use, selection by non-insecticidal compounds such as soap and hydrogen peroxide, affect adult mosquito tolerance to pyrethroids. With the current adaptation of mosquitoes to urban areas, it is important that such findings be taken in consideration by current and future control programmes.

## Competing interests

The authors declare that they have no competing interests.

## Authors’ contributions

Conceived and designed the study protocol: CAN, Participated in field and laboratory analyses, MYG, BTF, EK, PAA, CAN. Critically revised the manuscript: CC, NF, PAA. Interpreted, analysed data and wrote the paper: CAN. All authors read and approved the final version of the manuscript.
